# The effect of exercise complexity on trunk muscle activation and perceived exertion during body-weight training in recreationally trained adults

**DOI:** 10.3389/fspor.2026.1845995

**Published:** 2026-07-08

**Authors:** Vidar Andersen, Katarina Rise, Tor Einar Sandvikmoen, Tom Erik Jorung Solstad, Nicolay Stien, Thomas Bjørnsen, Suzanne Scott, Olaf Prieske, Atle Hole Saeterbakken

**Affiliations:** 1 Faculty of Education, Arts and Sports, Western Norway University of Applied Sciences, Sogndal, Norway; 2 Helsehuset Kristiansand, Kristiansand, Norway; 3 Department of Education and Sports Science, Faculty of Arts and Education, University of Stavanger, Stavanger, Norway; 4 School of Anatomy, University of Bristol, Bristol, United Kingdom; 5 Division of Exercise and Movement, University Applied Sciences for Sport and Management Potsdam, Potsdam, Germany

**Keywords:** bilateral, core training, EMG, instability, unilateral

## Abstract

**Introduction:**

The aim of the present study was to investigate the acute effects of varying exercise complexity on trunk muscle activity in recreationally trained adults during seven trunk-specific body-weight (BW) exercises.

**Methods:**

Twenty-eight participants were recruited (15 women and 13 men, age: 32 ± 9 years, height: 173 ± 9 cm, body mass: 73 ± 10 kg, training experience: 15 ± 9 years). Participants performed seven trunk-specific BW exercises in a randomized, counter-balanced order. Each exercise was performed isometrically (15 s) at 3–4 complexity levels, induced by changing the base of support, altering lever arms (using body tilt or body position), and applying bilateral versus unilateral execution. Electromyographic activity was collected from the rectus abdominis, external oblique, and spinal erector muscles. After completing each complexity level, participants rated their perceived exertion (RPE).

**Results:**

Muscle activation was significantly higher at larger complexity levels (*p* < 0.05, *η*_p_^2^ = 0.12–0.89), and this effect was most pronounced in the primary target muscles for each exercise. Higher levels of muscle activation were accompanied by higher levels of perceived exertion (*p* < 0.05, *w* = 0.19–0.90).

**Discussion:**

In conclusion, increasing instability, applying unilateral performance, and/or changing body position led to increased activation of trunk muscles and RPE during BW exercises in recreationally trained adults, with the highest activation observed in primary target muscles for each specific exercise.

## Introduction

The muscles commonly referred to as the core have been the subject of extensive scientific investigation over several decades (1, 2). Throughout this period, there have been different views regarding which muscles should be encompassed within the core construct (3). Bergmark (1) restricted the definition of core to muscles integrating the pelvis, spine, and rib cage, distinguishing between the deep (e.g., transversus, multifidus) and superficial muscles (e.g., rectus abdominis, erector spinae). Notably, Bergmark did not use the term core muscles, but instead used the more anatomical term trunk muscles. Other researchers have suggested a broader and more functional conceptualization, including muscles originating from or inserting into the extremities (e.g., the glutes and latissimus dorsi), provided they contribute to stabilizing the trunk or transferring forces to or from the trunk (4–6). Irrespective of the conceptual differences, the core/trunk may be understood as a complex system of muscles functioning to maintain the stability of the body and facilitate the transfer of kinetic energy across body segments—functions critical for athletes, recreationally trained individuals, and patients (7). In the present study, we adopted the more anatomical term trunk muscles over the more functional term core muscles. Furthermore, we operationalized the term to include the superficial abdominal and lower back muscles, that is, the rectus abdominis, external oblique, and erector spinae.

The trunk muscles have received much scientific attention in the contexts of physical fitness (8, 9), sport performance (10, 11), rehabilitation, and general health (12, 13). They can be stimulated by using different exercise approaches, such as free weights exercises (14, 15), or by using specially designed machines/equipment (16, 17). In addition, body-weight (BW) exercises, such as sit-ups and planks, are widely used for targeting the trunk muscles (18). In more traditional resistance training exercises there is a relationship between load and muscle activation, with an increase in electromyographic (EMG) activity observed as loading is increased (19–21). In contrast, controlling the intensity when BW is used as resistance is more challenging compared to free weights or machines. However, strategies such as changing lever arms (e.g., by modifying body position), executing the exercises unilaterally (e.g., single leg support in a pelvic bridge), or increasing stability demands (e.g., using balance pads) have been suggested to increase muscle activation and exercise intensity (22–24). It would be of scientific interest and practical value to explore the muscle activation in response to modifiers of intensity during specific trunk BW exercises, by investigating combinations of these different strategies (i.e., lever arm, stability demand, and unilateral loading bias).

In terms of body position, Moreno-Navarro et al. (23) compared the traditional prone plank (feet on the floor) with variations involving increased body tilt through foot elevation angle. The authors reported higher activation of the rectus abdominis, internal and external obliques, and the spinal erector muscles when the trunk was negatively angled (feet higher than the floor) compared to neutral (feet on the floor). These findings were partially supported by Yates et al. (25), who reported increased activation of external oblique and rectus abdominis muscles, but not the spinal erector muscle, when they compared three different negative inclinations of the trunk relative to the prone plank. The declined body tilt angle increased activation in the rectus abdominis by 28% and the external oblique by 17%, compared with the neutral plank position.

Furthermore, higher trunk muscle activation has been reported when exercises impose greater stability demands, such as by using unstable surfaces, unstable equipment, or reducing the base of support (22, 26–29). For example, Snarr et al. (26) reported higher activation of rectus abdominis and the spinal erector muscles when performing the pike on four different unstable surfaces compared with a stable surface, whereas no differences were observed between the different unstable conditions. Similarly, Panhan et al. (29) found higher activation of the m. rectus abdominis [84% vs. 55% of maximal voluntary contraction (MVC)] and m. internal oblique (92% vs. 57% of MVC) muscles during the Pilates exercise double leg stretch (i.e., bilateral lower limb trunk loading) when performed on a smaller base of support (small box compared to a mat).

Interestingly, some authors reported no difference or only limited differences during similar trunk loading protocols (30–32). Furthermore, others reported differences on only one side of the body (33, 34). For example, Ekstrom et al. (32) did not observe any differences in lower back (m. longissimus thoracis and m. lumbar multifidus) or abdominal (rectus abdominis and external oblique) muscles when comparing bilateral and unilateral bridges in healthy men (EMG data were only collected on one side of the trunk). In contrast, Feldwieser et al. (24) measured abdominal and lower back muscles bilaterally during different variations of the bridge (four bilateral and four unilateral). Importantly, the authors focused on differences between the left and right sides within each variation, and not on differences between the variations. The authors reported asymmetries in EMG between the left and right sides in three of the variations for the lower back muscles and in seven of the variations for the abdominal muscles.

Consequently, these findings highlight that multiple trunk muscles should be assessed bilaterally when comparing the acute effects of different executions of BW exercises. To the best of our knowledge, no studies have examined acute effects of varying exercise complexity—through a systematic combination of stability demand, base of support, trunk inclination, and/or unilateral execution—on bilateral trunk muscle myoelectric activity. Furthermore, none of the abovementioned studies measured the intensity, for example, using the rate of perceived exertion (RPE) of the different exercise variations. Therefore, the aim of the present study was to explore the effects of exercise complexity on trunk muscle activation and RPE during seven trunk-specific BW exercises in recreationally trained adults. The assessment was limited to the rectus abdominis, external oblique, and erector spinae muscles.

## Methods and materials

### Study design

**Figure 1:**
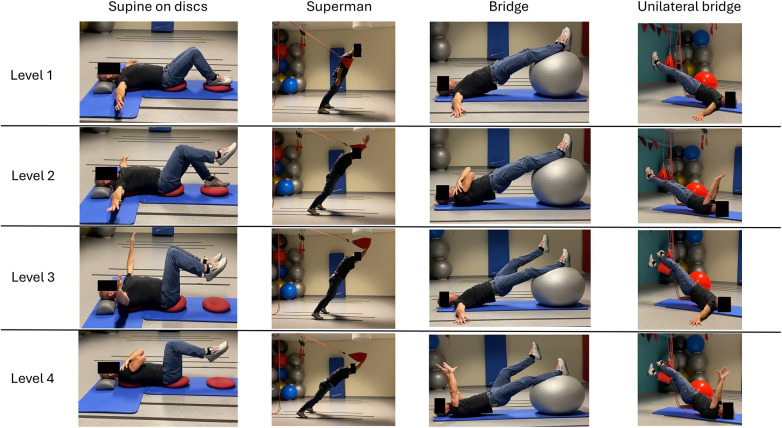
Different levels (1–4) of supine on inflatable discs, superman in slings, bridge on Swiss ball, and unilateral bridge in slings.

**Figure 2:**
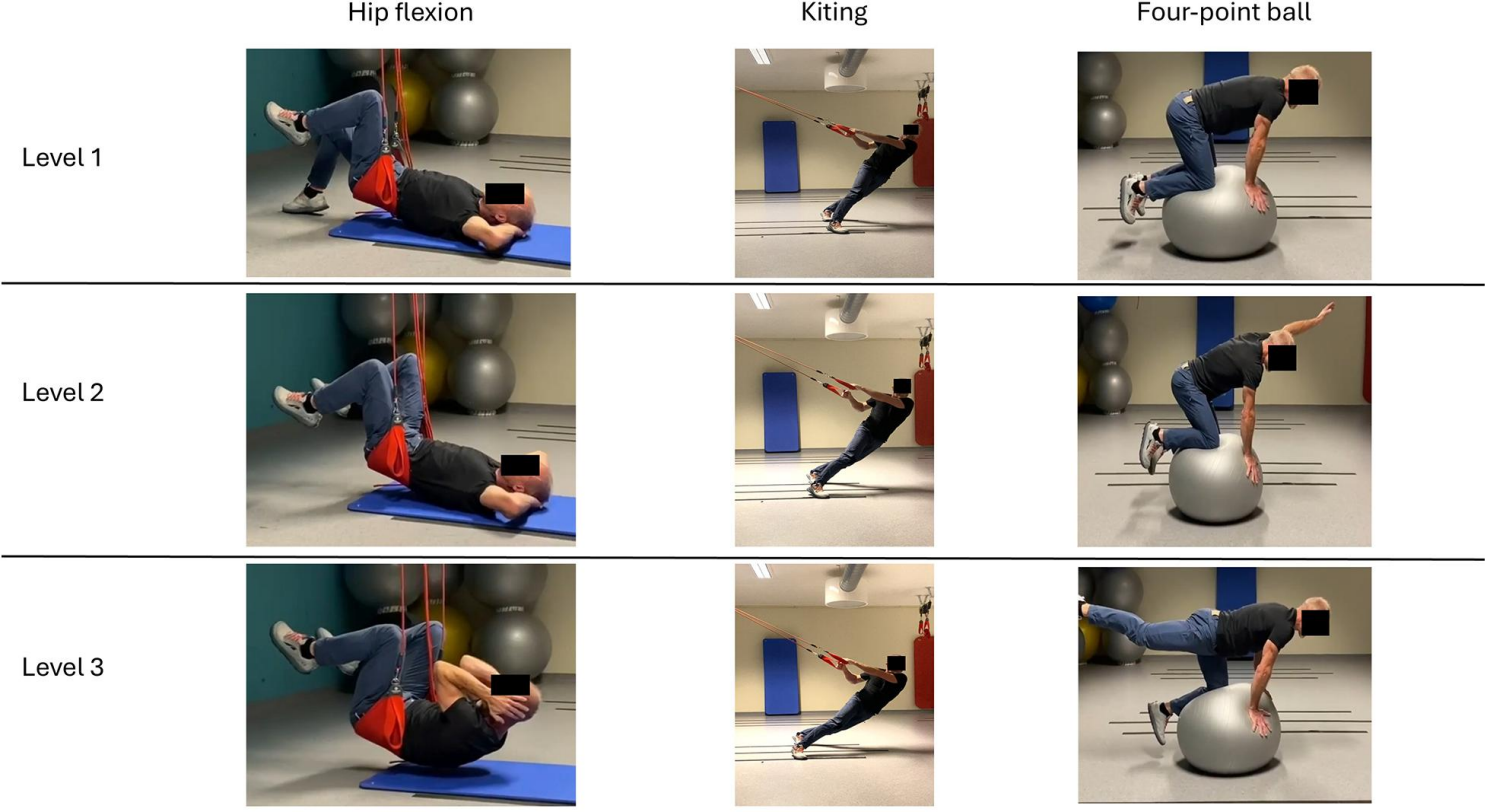
Different levels (1–3) of hip flexion in slings, kiting in slings, and four-point standing on Swiss ball.

This exploratory study used a within-subject crossover design to compare the superficial EMG activity of trunk muscles across varying complexity levels in seven trunk-specific BW exercises: supine on inflatable discs, superman in slings, bridge on Swiss ball, unilateral bridge in slings, hip flexion in slings, kiting in slings, and four-point standing on Swiss ball (see Table 1 and Figures 1, 2). The order of complexity levels for each exercise was randomized and counterbalanced. EMG activity was recorded from both sides of the trunk for the m. rectus abdominis, m. external oblique, and m. erector spinae. Modification of complexity level was achieved by changing the base of support, changing the lever arms (by trunk inclination and body position), and conducting the exercises bilaterally or unilaterally. At each complexity level, exercise position was isometrically held for 15 s. After completing exercises at each complexity level, participants' RPE was obtained. All experimental data were collected during the same session. A familiarization session was conducted 3–7 days before the experimental session to help participants become accustomed to the exercises and the RPE scale.

**Table 1:** Description of the execution of the different exercises and their complexity levels.

Name	Level 1	Level 2	Level 3	Level 4
Supine on inflatable discs	Lying supine with discs under scapulae, lower back, and feet (90° at the knee joint) and arms straight.	As level 1, but isometrically maintain right leg approximately horizontal (90–90 angle). Smaller base of support.	Trunk as level 1, but lift both legs (no ground contact), femurs vertical (90 degrees at the hip). Smaller base of support.	Trunk as level 3, but cross arms onto opposite shoulder. Smaller base of support.
Superman in slings	Standing on the toes, 80% of body length away from the anchor where the sling is attached, slings resting near the chest while leaning forward, legs and trunk aligned.	As level 1, but resting the slings on the elbows with elbows lifted and aligning with the legs and trunk. Increased lever arm.	As level 2, but holding the slings in the hands. Increased lever arm.	As level 3, but standing 60% of body length away from the anchor where the sling is attached. Increased lever arm.
Bridge on Swiss ball	Supine on mat, calves resting on Swiss ball, trunk aligned with the legs and arms out to the sides.	As level 1, but arms crossed and holding the opposite shoulder. Smaller base of support.	As level 1, but lifting one leg. Executed unilaterally.	As level 3, but holding arms vertically. Smaller base of support.
Unilateral bridge in slings	Supine on mat, right calf resting in sling, trunk and legs aligned, and arms straight and to the side.	As level 1, but holding arms vertically. Smaller base of support.	As level 1, but rotating one leg (e.g., left) toward the opposite side (e.g., right). Increased load.	As level 3, but hold arms vertically. Smaller base of support.
Hip flexion in slings	Lying supine on mat, sling under the lower back, pelvis elevated off the floor (approximately 10 centimeters), one leg (e.g., right) on the floor (90° at the knee), the other leg (e.g., left) lifted, femur vertical, and arms rest behind head. In this position, push the lower back down toward the sling as hard as possible.	As level 1, but with both legs lifted. Smaller base of support.	As level 2, but “crunching” (neck and trunk flexion) the upper body. Smaller base of support.	N/A
Kiting in slings	Standing 100% of body height away from the anchor where the sling is attached. Slings held in the hands while leaning forward and rotating to the right, toes pointing forward or slight external foot rotation, legs and trunk as aligned as possible.	As level 1, but 80% of body height away from the anchor where the sling is attached. Increased lever arm.	As level 1, but 60% of the body height away from the anchor where the sling is attached. Increased lever arm.	N/A
Four-point standing on Swiss ball	Standing on Swiss ball, both knees and hands in contact with the ball.	As level 1, but lift one arm (e.g., left) until horizontal. Smaller base of support.	As level 1, but lift one leg (e.g., right) until horizontal. Smaller base of support.	As level 1, but lift left arm and right leg until horizontal.^a^ Smaller base of support.

The primary justification for the increase in level (compared with the previous level) is provided at the end of each description.

N/A, not available.

a This modification was removed from the analysis because only six participants were able to perform this level.

#### Participants

To be recruited for the study, the participants had to be >18 years of age, physically active on a regular basis (train at least one session per week), and free from injuries or pain prohibiting exercise execution. An a priori sample size calculation was conducted, based on previous reported differences in muscle activity between left and right sides of the external oblique (9.9% ± 17.1%) and lower back muscles (11.7% ± 18.9%) when performing the bridge on a Swiss ball (24). The calculation was performed using SPSS (IBM Corp. Released 2022. IBM SPSS Statistics for Windows, Version 29.0. Armonk, NY: IBM Corp). With an alpha level of 0.05 and statistical power of 80%, the required sample size to detect a meaningful effect was 23–26 participants (ES < 0.58). Subjects were recruited via social media, flyers, and information meetings. A total of 28 volunteers (15 women, 13 men) were enrolled. Participants (age: 32 ± 9 years, height 173 ± 9 cm, body mass 73 ± 10 kg) reported a training experience of 15 ± 9 years. Their weekly training schedule consisted of 3.9 h of endurance training and 2.7 h of resistance training. All participants were informed orally and in writing about the project before providing their written consent to be enrolled. The study procedure was approved by the Norwegian Centre for Research Data (Ref: 648519) and the University College Ethical Committee (Ref: 24/06917-3).

#### Procedures

Familiarization included (1) anthropometric data collection, (2) familiarization with the different exercises and complexity levels, and (3) familiarization with the RPE scale. The order of the exercises and complexity levels was randomized and counterbalanced; however, the same order was maintained for each participant across both the familiarization and experimental sessions. For each exercise, progression of complexity level was based on the instructor's (TorS) extensive professional experience as a physiotherapist working with both athlete and patient populations.

Seven exercises were included: supine on inflatable discs, superman in slings, bridge on Swiss ball, unilateral bridge in slings, hip flexion in slings, kiting in slings, and four-point standing on Swiss ball. Descriptions of the exercises and their different complexity levels are provided in Table 1 and illustrated in Figures 1, 2.

The position for each level was held statically (i.e., isometric contraction) for 15 s. The rest interval between each level was 90 s and between each exercise was 120 s. Participants had to complete all levels of an exercise to be included in the analyses for that specific exercise.

#### Assessment of muscle activity

Surface EMG amplitude reflects the electrical excitation of muscle fibers at the skin surface (i.e., myoelectric activity), and is related to, but not a direct measure of, motor unit recruitment, discharge rates, and force production (35). In this study, the term “muscle activation” was used as conventional shorthand to denote normalized surface EMG amplitude.

To measure neuromuscular activation, surface EMG was recorded using a synchronized unit (MuscleLab 6,000 system) and analyzed using MuscleLab software (Ergotest Technology AS, Porsgrunn, Norway). Before attaching the electrodes, the skin was shaved, abraded, and washed with alcohol, according to previous recommendations (36). Gel-coated self-adhesive electrodes (a 2-cm center-to-center distance, with an 11-mm contact diameter) were placed on both sides of the trunk in the direction of underlying fibers of the target muscles (i.e., m. erector spinae, m. rectus abdominis, and m. obliquus external externus). Electrode placement was according to SENIAM guidelines (http://seniam.org). More precisely, the electrodes on the spinal erector muscle were placed two finger widths lateral to the spinous process of the first lumbar vertebra; on the rectus abdominis muscle, they were placed 3 cm lateral to the umbilicus; and for the external oblique, they were placed approximately 15 cm lateral to the umbilicus. To minimize noise from external sources, raw EMG signals were amplified and filtered using a preamplifier (input impedance; 1,000 GΩ) located close to the sampling point. The common-mode rejection ratio of the preamplifier was 106 dB, with a fourth-order Butterworth band pass filter (high-cut frequency of 500 Hz and low-cut frequency of 20 Hz). The EMG signals were sampled at a rate of 1,000 Hz as well as rectified, integrated, and converted to root mean square (RMS) EMG signals. The mean RMS signal for each muscle was recorded over a 15-s period for each exercise and difficulty level, with 2.5 s at the beginning and at the end of the exercise being removed. The remaining 10 s were normalized to the participants' maximal isometric voluntary contraction (MIVC) for each muscle before being used in further analyses. Two trials were conducted for MIVCs, with a rest interval of 90 s between each attempt. For the abdominal muscles, two isometric sit-ups were used [straight for the rectus abdominis muscle and diagonal (both sides) for the external oblique muscles], where participants were held in a sitting position with hip and knee at an angle of 90°. For the spinal erector muscles, participants lay prone on the floor with their lower body fixed. Participants were instructed to extend their back while manually being held in a static position. During all MIVC trials, participants were instructed to work at maximal force and hold for ∼3 s. Two attempts were performed for each test and the one with the highest EMG amplitude was used to normalize the EMG signal for each muscle. The average value of the normalized EMG data for both sides of the same muscle at the same level (left and right sides of rectus abdominis, external oblique, and the erector spinae) was used in analyses.

#### Assessment of ratings of perceived exertion

Immediately after completing each exercise level, participants were asked to rate their perceived exertion for effort using the Borg CR10 scale. The scale consists of 11 points, ranging from “no effort” to “maximal effort.” The scale was presented to the participants (both shown and read) with the following statement: “How much of your perceived physical capacity out of your perceived maximum (10 being your maximum) did you use during this exercise?” The upper and lower limits were anchored by the following sentence: “0 can be described as sitting still during the whole exercise, while 10 would be maximal effort, using your maximal physical capacity throughout the whole exercise.”

#### Statistical analysis

All statistical analyses were performed using SPSS (IBM Corp. Released 2020. IBM SPSS Statistics for Windows, Version 29.0. Armonk, NY: IBM Corp). The normal distribution of the EMG data was confirmed by visual inspection. To examine the effect of exercise complexity on EMG activation, an analysis of variance with repeated measures (rmANOVA) was conducted on factor complexity level. Mauchly’s sphericity test was used to check for homogeneity of variances. The Greenhouse–Geisser correction was applied when homogeneity was violated. When a main effect was detected, paired t-tests with Bonferroni *post hoc* correction were performed to identify the different conditions. Effect sizes for the rmANOVA tests are presented as partial eta squared (*η*_p_^2^). Values between 0.01 and 0.06 were considered small, between 0.06 and 0.14 medium, and above 0.14 large (37). Effect sizes for the *post hoc* comparisons are presented as Cohen’s *d* effect size (*d*) and calculated using the following equation: mean level A – mean level B divided by the standard deviations of the difference. An effect size of 0.2–0.5 was considered small, 0.5–0.8 medium, and >0.8 large (37). These results are presented as mean ± standard deviation (SD).

For the RPE data, the Friedman test was used to examine if RPE varied according to the difficulty level. If differences were detected, the Wilcoxon signed-rank test was used to identify where the differences lay. To avoid potential type I error due to multiple comparisons, the *p*-value from the Wilcoxon signed-rank test was multiplied by the number of comparisons (Bonferroni correction). For the Friedman test, effect size was calculated as Kendall’s W (*w*) value by the following equation: *w* = *x*2/*N*(*K*-1) (*x*2 being Friedman test statistic value, *N* the number of participants, and *K* the number of measurements per participant). An effect size of 0.2–0.5 was considered small, 0.5–0.8 medium, and >0.8 large (37). For the Wilcoxon signed-rank test the effect size was calculated as product-movement *r* (*r*) using the following equation: *r* =* z*/√*n* (*z* being the *z*-value of the Wilcoxon signed-rank test and *n* being the number of participants). A product movement *r* of 0.1–0.29 was considered small, 0.3–0.49 medium, and ≥0.5 large (37). These data are presented as median ± interquartile range. For all data, statistical significance was set at *p* < 0.05.

## Results

The EMG and RPE data, together with the rmANOVA results, are presented in Table 2.

**Table 2:** Electromyographic activity (% of MIVC given as mean ± standard deviation) and rating of perceived exertion (given as median ± interquartile range) for the different levels and exercises.

Muscle/RPE	Level 1	Level 2	Level 3	Level 4	Main effect
Supine on inflatable discs (*n* = 28)					
External oblique	10.4 ± 7.5^2–4^	21.5 ± 14.0^3–4^	36.9 ± 18.4	36.3 ± 19.7	*p* = <0.01, *η*_p_^2^ = 0.55
Erector spinae	9.8 ± 7.5^2–3^	15.9 ± 12.5	16.3 ± 14.2	14.1 ± 13.2	*p* = 0.01, η_p_^2^ = 0.15
Rectus abdominis	7.8 ± 11.4^3–4^	14.1 ± 16.6^3^	22.9 ± 15.4	24.8 ± 23.2	*p* = <0.01, η_p_^2^ = 0.31
RPE	2 ± 1^2–4^	3 ± 2^4^	4 ± 3	5 ± 3	*p* = <0.01, w = 0.68
Superman in slings (*n* = 27)					
External oblique	11.9 ± 9.6^3–4^	13.9 ± 9.9^3–4^	28.6 ± 20.5^4^	40.1 ± 25.8	*p* = <0.01, η_p_^2^ = 0.57
Erector spinae	12.1 ± 8.5^2–3^	8.9 ± 7.2	7.5 ± 6.1^4^	9.3 ± 7.1	*p* = 0.01, η_p_^2^ = 0.20
Rectus abdominis	7.1 ± 7.8^3–4^	10.1 ± 15.3^3–4^	23.6 ± 19.5^4^	39.3 ± 29.0	*p* = <0.01, η_p_^2^ = 0.53
RPE	1 ± 1^3–4^	1 ± 1^3–4^	3 ± 2^4^	4 ± 2	*p* = <0.01, w = 0.90
Bridge on Swiss ball (*n* = 26)					
External oblique	10.5 ± 8.1^3–4^	14.0 ± 16.8^3–4^	21.3 ± 17.9^4^	29.5 ± 22.2	*p* = <0.01, η_p_^2^ = 0.48
Erector spinae	42.1 ± 23.1	42.6 ± 24.6	48.4 ± 30.0	49.6 ± 28.5	*p* = 0.05, η_p_^2^ = 0.13
Rectus abdominis	7.1 ± 8.0^4^	6.9 ± 9.0^4^	8.8 ± 8.1	16.0 ± 19.3	*p* = <0.01, η_p_^2^ = 0.31
RPE	2 ± 2^2–4^	3 ± 2^3–4^	4 ± 2^4^	5 ± 2	*p* = <0.01, w = 0.87
Unilateral bridge in slings (*n* = 27)					
External oblique	21.6 ± 16.8	19.6 ± 14.6	26.8 ± 16.8	22.9 ± 16.2	*p* = 0.03, η_p_^2^ = 0.12
Erector spinae	46.6 ± 20.0	43.7 ± 16.1	45.3 ± 18.8	44.9 ± 18.7	*p* = 0.35, η_p_^2^ = 0.04
Rectus abdominis	10.5 ± 13.7	10.2 ± 13.5	11.7 ± 14.7	10.7 ± 13.6	*p* = 0.32, η_p_^2^ = 0.04
RPE	4 ± 2^4^	4 ± 1^4^	4 ± 2	5 ± 2	*p* = <0.01, w = 0.19
Hip flexion in slings (*n* = 28)					
External oblique	46.0 ± 25.4^2–3^	66.6 ± 33.3^3^	92.5 ± 42.8		*p* = <0.01, η_p_^2^ = 0.55
Erector spinae	10.1 ± 5.7	12.7 ± 7.9	14.1 ± 10.7		*p* = 0.05, η_p_^2^ = 0.13
Rectus abdominis	42.8 ± 26.5^2–3^	60.4 ± 20.9^3^	83.2 ± 36.5		*p* = <0.01, η_p_^2^ = 0.44
RPE	5 ± 2^2–3^	5 ± 2^3^	6 ± 2		*p* = <0.01, w = 0.36
Kiting in slings (*n* = 24)					
External oblique	23.7 ± 14.6^2–3^	30.1 ± 17.2^3^	41.7 ± 23.6		*p* = <0.01, η_p_^2^ = 0.54
Erector spinae	14.6 ± 12.0^3^	18.0 ± 14.4^3^	25.6 ± 18.8		*p* = <0.01, η_p_^2^ = 0.33
Rectus abdominis	11.4 ± 10.6^3^	16.0 ± 18.4	22.1 ± 24.0		*p* = <0.01, η_p_^2^ = 0.28
RPE	2 ± 1^2–3^	3 ± 2^3^	5 ± 2		*p* = <0.01, w = 0.89
Four-point standing on Swiss ball (*n* = 27)					
External oblique	20.1 ± 15.3^3^	31.8 ± 28.9	43.2 ± 26.7		*p* = <0.01, η_p_^2^ = 0.29
Erector spinae	16.1 ± 19.1^2–3^	34.0 ± 27.5	30.5 ± 35.2		*p* = <0.01, η_p_^2^ = 0.36
Rectus abdominis	16.7 ± 16.1^3^	17.1 ± 20.0^3^	22.7 ± 20.9		*p* = 0.01, η_p_^2^ = 0.16
RPE	3 ± 1^2–3^	4 ± 2	5 ± 3		*p* = <0.01, w = 0.69

2–4 = sign, different *p* < 0.05 from levels 2–4; 3 and 4 = sign, different *p* < 0.05 from levels 3 and 4; 2 and 3 = sign, different *p* < 0.05 from levels 2 and 3; 3 = sign, different *p* < 0.05 from level 3; and 4 = sign, different *p* < 0.05 from level 4. RPE = rating of perceived exertion.

### Supine on inflatable discs

Significant main effects were found for all muscles with large effect sizes (Table 2).

For the m. external oblique, * post hoc* tests showed that EMG activation at level 1 was significantly lower than all other levels (*p* = <0.01–0.01, *d* = 0.99–1.89). Level 2 was significantly lower than levels 3 and 4 (*p* = <0.01–0.01, *d* = 0.86–0.94). There was no significant difference between levels 3 and 4 (*p* = 1.00, *d* = 0.03).

For the m. erector spinae, * post hoc* corrections showed that for level 1, EMG activation was significantly lower than levels 2 and 3 (*p* = <0.01–0.02, *d* = 0.57–0.59). There were no significant differences among the other levels (*p* = 0.27–1.00, *d* = 0.03–0.40).

For the m. rectus abdominis, *post hoc* tests showed that for level 1, EMG activation was significantly lower than levels 3 and 4 (*p* = <0.01, *d* = 0.93–1.12). Level 2 was significantly lower than level 3 (*p* = 0.01, *d* = 0.55), with no other differences among the other levels (*p* = 0.14–1.00, *d* = 0.53–0.10).

The Friedman test revealed a significant main effect for RPE between levels (Table 2). The Wilcoxon signed-rank test demonstrated that level 1 was rated significantly lower than levels 2–4 (*p* = <0.01, *r* = 0.82–0.86) and level 2 rated significantly lower than level 4 (*p* = <0.01, *r* = 0.69). There were no significant differences between levels 2 and 3 (*p* = 0.07, *r* = 0.48) or levels 3 and level 4 (*p* = 0.29, *r* = 0.42).

### Superman in slings

Main effects were observed for all muscles with large effect sizes (Table 2).

For the m. external oblique, *post hoc* corrections showed that activation at levels 1 (*p* = <0.01, *d* = 1.04–1.45) and 2 (*p* = <0.01, *d* = 0.92–1.35) was significantly lower than levels 3 and 4. Level 3 was significantly lower than level 4 (*p* = <0.01, *d* = 0.49). There was no difference between level 1 and 2 (*p* = 1.00, *d* = 0.20).

For the m. erector spinae, EMG activation recorded at level 1 was significantly greater than levels 2 and 3 (*p* = 0.01–0.03, *d* = 0.40–0.61). Level 4 was greater than level 3 (*p* = <0.01, *d* = 0.28), with no other significant differences between the other levels (*p* = 0.24–1.00, *d* = 0.06–0.35).

For the m. rectus abdominis, *post hoc* tests revealed that for levels 1 (*p* = <0.01, *d* = 1.10–1.52) and 2 (*p* = <0.01, *d* = 0.77–1.23), EMG activation was significantly lower than levels 3 and 4. Activation at level 3 was lower than at level 4 (*p* = <0.01, *d* = 0.64). There was no difference between levels 1 and 2 (*p* = 1.00, *d* = 0.25).

The Friedman test demonstrated a significant main effect for RPE across the levels (Table 2). The Wilcoxon signed-rank test demonstrated a significantly lower RPE in levels 1 (*p* = <0.01, *r* = 0.86–0.88) and 2 (*p* = <0.01, *r* = 0.81–0.88) compared to levels 3 and 4. Level 3 was rated significantly lower than level 4 (*p* = <0.01, *r* = 0.79. There was no significant difference between levels 1 and 2 (*p* = 0.29, *r* = 0.38).

### Bridge on Swiss ball

A main effect for EMG activation was observed for the m. external oblique and m. rectus abdominis, with large effect sizes. However, there was no main effect for the m. erector spinae (Table 2).

For the m. external oblique, *post hoc* corrections showed that EMG activation at levels 1 (*p* = <0.01, *d* = 0.78–1.13) and 2 (*p* = <0.01, *d* = 0.43–0.79) was significantly lower than at levels 3 and 4. Activation at level 3 was lower than at level 4 (*p* = <0.01, *d* = 0.40). There was no difference between levels 1 and 2 (*p* = 1.00, *d* = 0.26).

For the m. rectus abdominis, *post hoc* tests demonstrated that for levels 1 (*p* = 0.01, *d* = 0.61) and 2 (*p* = 0.01, *d* = 0.60), EMG activation was significantly lower than level 4, with no other differences found between levels (*p* = 0.06–1.00, *d* = 0.14–0.49).

For the RPE, a significant main effect of level was observed (Table 2). The Wilcoxon signed-rank test revealed a progressive step-wise increase in intensity. In particular, level 1 was perceived as easier than all other levels, (*p* = <0.01, *r* = 0.78–0.86), while level 2 was perceived as easier than levels 3 (*p* = <0.01, *r* = 0.81) and 4 (*p* = <0.01, *r* = 0.82). Level 3 was perceived as easier than level 4 (*p* = <0.01, *r* = 0.68).

### Unilateral bridge in slings

The only main effect for this exercise was found in the m. external oblique (Table 2); however, *post hoc* tests revealed no significant differences between the levels (*p* = 0.05–1.00, *d* = 0.07–0.46).

The Friedman test revealed a main effect for RPE between the different levels (Table 2). The Wilcoxon signed-rank test demonstrated that RPE was rated significantly lower for level 1 compared with level 4 (*p* = 0.04, *r* = 0.52) and for level 2 compared with level 4 (*p* = 0.03, *r* = 0.44). There were no significant differences between the other levels (*p* = 0.13–1.00, *r* = 0.20–0.54).

### Hip flexion in slings

Significant main effects were found for the m. external oblique and m. rectus abdominis, with large effect sizes; however, there was no main effect for the m. erector spinae (Table 2).

For the m. external oblique, *post hoc* tests revealed that for level 1, EMG activation was significantly lower than at levels 2 and 3 (*p* = <0.01, *d* = 0.69–1.32). Activation at level 2 was significantly lower than at level 3 (*p* = <0.01, *d* = 0.67).

For the m. rectus abdominis, *post hoc* tests showed that activation at level 1 was significantly lower than at levels 2 and 3 (*p* = <0.01, *d* = 0.74–1.27). Activation at level 2 was significantly lower than at level 3 (*p* = <0.01, *d* = 0.77).

There was a main effect for RPE between the different levels (Table 2). The Wilcoxon signed-rank test revealed that perceived exertion was lower at level 1 compared with level 2 (*p* = 0.01, *r* = 0.49) and level 3 (*p* = <0.01, *r* = 0.69). Exertion in level 2 was lower compared with level 3 (*p* = <0.01, *r* = 0.62).

### Kiting in slings

Main effects were observed for EMG activation in all muscles, with large effect sizes (Table 2).

For the m. external oblique, *post hoc* tests demonstrated that activation at level 1 was significantly lower than at levels 2 and 3 (*p* = <0.01, *d* = 0.40–0.92). Activation at level 2 was significantly lower than at level 3 (*p* = <0.01, *d* = 0.56).

For the m. erector spinae, *post ho* -corrections showed that level 1 (*p* = <0.01, *d* = 0.69) and level 2 (*p* = <0.01, *d* = 0.45) were significantly lower than level 3, with no difference between levels 1 and 2 (*p* = 0.46, *d* = 0.25).

For the m. rectus abdominis, *post hoc* tests revealed that activation at level 1 was significantly lower than at level 3 (*p* = 0.01, *d* = 0.58); however, no difference was found between levels 1 and 2 (*p* = 0.15, *d* = 0.31) or levels 2 and 3 (*p* = 0.06, *d* = 0.29).

The Friedman test demonstrated a main effect for RPE between the different levels (Table 2). The Wilcoxon signed-rank test revealed that perceived exertion was significantly lower for level 1 compared with level 2 (*p* = <0.01, *r* = 0.89) and level 3 (*p* = <0.01, *r* = 0.86), and for level 2 compared with level 3 (*p* = <0.01, *r* = 0.75).

### Four-point standing on Swiss ball

Significant main effects were found for EMG activation according to level for all muscles, with large effect sizes (Table 2).

For the m. external oblique, *post hoc* tests demonstrated that at level 1, EMG activation was significantly lower than at level 3 (*p* = <0.01, *d* = 1.06). No differences were found between levels 1 and 2 (*p* = 0.09, *d* = 0.51) or levels 2 and 3 (*p* = 0.27, *d* = 0.41).

For the m. erector spinae, *post hoc* tests showed that activation at level 1 was significantly lower than at levels 2 (*p* = <0.01, *d* = 0.76) and 3 (*p* = <0.01, *d* = 0.51). No significant difference was observed between levels 2 and 3 (*p* = 1.00, *d* = 0.11).

For the m. rectus abdominis, *post hoc* tests revealed that at level 1 (*p* = 0.04, *d* = 0.32) and level 2 (*p* = 0.01, *d* = 0.28), EMG activation was significantly lower than at level 3, with no difference between levels 1 and 2 (*p* = 1.00, *d* = 0.02).

There was a main effect for RPE between the different levels (Table 2). The Wilcoxon Signed-Rank test demonstrated that RPE was significantly lower for level 1 compared to levels 2 (*p* = <0.01, *r* = 0.81) and3 (*p* = <0.01, *r* = 0.81). However, there was no significant difference between levels 2 and level 3 (*p* = 0.28, *r* = 0.32).

## Discussion

The main finding of the present study was that trunk muscle activation (in six out of seven exercises) and RPE (in all seven exercises) increased when complexity level was varied for trunk-specific BW exercises by increasing instability requirement, reducing the base of support, increasing trunk inclination, and/or performing exercises unilaterally.

Performing trunk-specific BW exercises at different complexity levels revealed a pattern of higher muscle activation with greater complexity level, although this effect was not significant across all levels and exercises. Similar to the EMG data, there was a pattern of increased ratings of perceived exertion as the complexity level of the exercises was increased. This finding is in line with previous studies reporting a positive trend for RPE and EMG to increase with increased resistance (20, 21). Notably, the RPE did not exceed a median score of 6 (level 3 in hip flexion in slings), which may be explained by the relatively brief (15) sec contraction (i.e., work) duration, which was deliberately chosen over longer durations or multiple contractions to avoid fatigue. Importantly, and in accordance with previous recommendations (35), all data were collected in the same session to ensure validity of the EMG data.

Notably, there are some exceptions from the interrelation between increases in complexity, EMG, and RPE, for example, between levels 3 and 4 in lying on infatable discs and between levels 1 and 2 in bridge on Swiss ball. A common feature of these exercises was that complexity was increased by reducing the base of support of the shoulders and arms. It is possible that this method of increasing intensity is insufficient to elevate muscle activation, at least in a population of recreationally trained individuals. Similarly, the lack of increased trunk muscle activation observed during the superman in slings at levels 1 and 2 could have resulted from insufficient intensity ramping for this specific exercise: i.e., that the lever arm has to be increased more than from chest to the elbows in order to elicit increased EMG activity.

The unilateral bridge in slings stands out from the rest of the exercises as the only exercise where level of complexity did not influence trunk muscle activation significantly. Notably, there was a main effect for the external oblique, but * post hoc* corrections did not reveal any statistically significant differences with increasing complexity. This finding may be explained by the exercise demand being similar in intensity at all levels of execution. The RPE results indicated that level 1 was perceived as a similar high demand compared to the other levels (RPE = 4). The fact that level 1 was quite demanding is supported by fairly high levels of muscle activation in the erector spinae and external oblique, when compared to level 1 activity for the other exercises. Furthermore, the increase in complexity was created by rotating the free leg (non-weight-bearing leg) and reducing the base of support for the shoulders and arms. As already mentioned, the latter method appears to be ineffective in ramping intensity, or at best only increases it insignificantly in recreationally trained individuals. Rotation of the non-weight-bearing leg was not used to ramp intensity in any other exercises; however, based on the analysis of this exercise, this approach has limited influence on trunk muscle activation.

Another explanation for the lack of pattern between increasing exercise complexity and increased trunk muscle activation is the insignificant involvement of the muscle in that specific exercise. For example, the erector spinae in superman in slings, the rectus abdominis in unilateral bridge in slings and the erector spinae in hip flexion in slings were not influenced by an increase in complexity level. A common denominator for these muscles is generally low levels of activation, implying that they should be regarded as antagonists rather than agonists for those specific exercises. Consequently, increasing the intensity by progressing to a higher level of that particular exercise does not necessarily affect the recruitment of that specific muscle. This finding implies that the trunk muscles should not be regarded as a holistic group, but rather as individual muscles. Furthermore, this should be an important consideration when designing training programs; that is to say, exercises should be selected based on the particular muscles one would like to stimulate.

This conclusion is supported by previous studies (14, 26, 28). For example, Czaprowski et al. (28) reported abdominal muscle activation of ∼2%–9% of MVC when the bridge was performed supine compared with ∼18%–55% of MVC when the bridge was performed prone. Moreover, Saeterbakken et al. (14) compared trunk muscle activation when performing squat and leg press with heavy loads (3-RM) and reported abdominal muscle activation (rectus abdominis and external oblique) at ∼12%–20%, whereas erector spinae activation reached ∼65%–80%. These results, together with the findings of the present study, emphasize how the muscle activity is highly dependent on the chosen exercise, and within each exercise, trunk muscle activation can vary across individual/specific muscles.

The present study has several limitations that should be acknowledged. For instance, surface EMG provides only an estimate of neuromuscular activation and carries a possible risk of crosstalk from nearby muscles (35, 38). Importantly, all EMG data were collected in the same session, which substantially reduces the potential for error associated with electrode placement (35). Next, trunk muscle activation was limited to the rectus abdominis, external oblique, and erector spinae. Hence, our findings cannot be generalized to other trunk muscles, such as the transversus abdominis and multifidus. Furthermore, only recreationally trained adults were recruited in this study, and the findings may not necessarily generalize to other populations (e.g., adolescents, sedentary older adults). Unilateral exercises/levels were only performed on one side, and we did not examine differences between the left and right sides of the trunk muscles. The reasons for this approach was to avoid fatigue and align with our primary research focus, as examining differences between sides was not our main research question. Future studies should investigate if and how unilateral execution affects activation on the different sides of the trunk muscles. In addition, each exercise and level was only performed once, which may have masked intraindividual variability, particularly given the different degrees of instability. Importantly, all participants completed a familiarization session prior to the experimental session, but since we did not record EMG in this session, we cannot ignore the possibility of bias from random variation. Finally, progression in complexity levels in the present study was based on combinations of increasing moment or lever arms by changing body position and/or decreasing the stability of the exercises. Thus, it is challenging to quantify the exact variations in difficulty between the exercise levels. However, we chose commonly applied variations of exercises that are widely used in practice to increase intensity within training programs.

## Conclusions

In conclusion, higher complexity levels in trunk-specific BW exercises—achieved through greater instability, performing exercises unilaterally, and/or changing body position—generally resulted in greater trunk muscle (rectus abdominis, external oblique, and erector spinae) activation as well as RPE in recreationally trained adults. However, increased muscle activity was particularly evident in the prime movers of the each exercise. These findings suggest that practitioners, athletes, coaches, and therapists should carefully consider both exercise selection and complexity level based on the athlete or patient's strength level and the target muscle of interest. Future studies should investigate the long-term effects of progressive resistance training interventions focusing on BW exercises to enhance strength of the different trunk muscles.

## Data Availability

The raw data supporting the conclusions of this article will be made available by the authors, without undue reservation.
